# Pneumococcal meningitis secondary to otitis media in two patients with COVID-19 omicron variant

**DOI:** 10.1186/s12245-022-00448-y

**Published:** 2022-09-14

**Authors:** Diego Franch-Llasat, Naya Bellaubí-Pallarés, Mar Olga Pérez-Moreno, Elena Chamarro-Martí, Esther García-Rodríguez, Ferran Roche-Campo

**Affiliations:** 1 Hospital de Tortosa Verge de la Cinta, Tarragona, Spain; 2 The Pere Virgili Institute for Health Research (IISPV), Reus, Spain

**Keywords:** Pneumococcal meningitis, Streptococcus pneumoniae, Otitis media, COVID-19, SARS-CoV-2, Omicron variant, B.1.1.529

## Abstract

**Background:**

The SARS-CoV-2 omicron variant produces more symptoms in the upper respiratory tract than in the lower respiratory tract. This form of “common cold” can cause inflammation of the oropharynx and the Eustachian tube, leading to the multiplication of bacteria such as Streptococcus pneumoniae in the oropharynx. Eustachian tube dysfunction facilitates migration of these bacteria to the middle ear, causing inflammation and infection (otitis media), which in turn could lead to further complications such as acute mastoiditis and meningitis.

**Case presentation:**

In January 2022, during the rapid spread of the omicron variant of the SARS-CoV-2 virus, two patients presented to the emergency room at our hospital complaining of headache and a low level of consciousness. A few days prior to admission, the patients had been diagnosed with COVID-19 based on clinical manifestations of a cold virus, without respiratory failure. Cranial computed tomography revealed signs of bilateral invasion of the middle ear in both cases. Lumbar puncture was compatible with acute bacterial meningitis, and S. pneumoniae was isolated in cerebrospinal fluid in both patients. RT-PCR tests for SARS-CoV-2 were repeated, confirming the presence of the omicron variant in one of the patients. We were unable to confirm the variant in the second patient due to the low viral load in the nasopharyngeal sample obtained at admission. However, the time of diagnosis (i.e., during the peak spread of the omicron variant), strongly suggest the presence of the omicron variant. Both patients were admitted to the intensive care unit and both showed rapid clinical improvement after initiation of antibiotic treatment.

**Conclusions:**

The omicron variant of the SARS-CoV-2 virus can promote the development of otitis media and secondary acute bacterial meningitis. S. pneumoniae is one of the main bacteria involved in this process.

## Background

Several respiratory viruses can predispose the host to secondary bacterial infections. The synergistic relationship between the influenza virus and bacterial infections (particularly *Streptococcus pneumoniae*) has probably received the most attention [1]. This virus alters the functions of the host’s epithelial barrier, modifying the innate and adaptive immune response and inducing changes in the microenvironment of the respiratory tract. *S. pneumoniae* takes advantage of these alterations to switch from colonization to infection. Although the association between influenza and bacterial infection has been extensively described, little is known about the possible association between the SARS-CoV-2 virus, which causes COVID-19, and the development of bacterial infections.

Here, we report two cases of patients with SARS-CoV-2 who required admission to the intensive care unit (ICU) due to acute bacterial meningitis secondary to otitis media. Both cases occurred during a single week in January 2022 when the omicron variant was spreading rapidly. The causal microorganism in both cases was *S. pneumoniae*.

The presence of the omicron variant (B.1.1.529) was confirmed by real time–polymerase chain reaction (RT-PCR) in one patient. Although we were unable to isolate the same variant in the other patient, it is highly likely that the same variant was involved. Compared to the original strain and prior variants, the omicron variant of SARS-CoV-2 has a greater impact on the upper respiratory tract than on the lower respiratory tract [2], which explains why neither patient developed acute respiratory failure. As we discuss below, involvement of the oropharynx promotes the development of otitis media.

## Case presentation

### Case 1

**Fig. 1:**
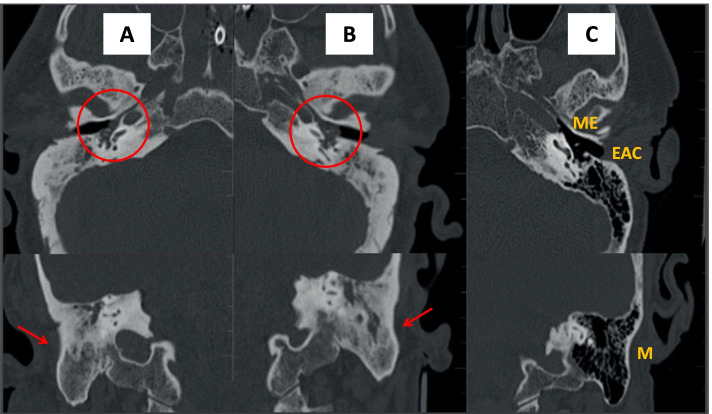
Computed tomography (CT) image of pathological petrous bone [**A** = right; **B** = left] compared to a normal CT (**C**). Axial images (superior) and coronal images (inferior). Bilateral invasion of the middle ear (red circle), mastoid cavity, and mastoid air cells (red arrow)

A 61-year-old male with a history of type II diabetes mellitus and high blood pressure. The patient had been immunized against SARS-CoV-2 (two doses), but not against *S. pneumoniae*. On January 1, 2022 he developed symptoms compatible with the common cold; the next day, he tested positive (RT-PCR) for SARS-CoV-2. Four days later (January 6), the patient reported headache and pain in the left ear. On January 7, he presented at the emergency department due to a low level of consciousness (Glasgow coma scale [GCS], 7 points). Cranial computed tomography (CT) showed signs of invasion in the left middle ear. Lumbar puncture was compatible with acute bacterial meningitis. RT-PCR analysis of cerebrospinal fluid (CSF) was positive for *S. pneumoniae* and the patient was admitted to the ICU. Orotracheal intubation was performed and the patient was put on mechanical ventilation. The patient again tested positive for SARS-CoV-2, but with a low viral load. The presence of *S. pneumoniae* was isolated in blood cultures and treatment with ceftriaxone was started. A second cranial CT scan was performed, revealing bilateral otomastoiditis (Fig. 1). The neurological symptoms progressively improved and the patient was discharged from the ICU on January 19.

### Case 2

A 61-year-old woman with no past medical history of note. She had been immunized against SARS-CoV-2 (two doses), but not against *S. pneumoniae*. On January 5, 2022, she developed cold symptoms and the next day tested positive (RT-PCR) for SARS-CoV-2. Four days later (January 10), the patient reported pain in the right ear. The next day, she presented to the emergency department with a low level of consciousness (GCS, 11). Cranial CT revealed diffuse cerebral edema and bilateral invasion of the middle ear. Lumbar puncture was compatible with acute bacterial meningitis. Subsequently, CSF culture was positive for *S. pneumoniae*. The RT-PCR remained positive for SARS-CoV-2 and the omicron variant was identified through next-generation sequencing (NGS). The patient was admitted to the ICU. Orotracheal intubation was not required. Treatment with ceftriaxone was started and the patient’s clinical status improve rapidly. She was discharged from the ICU on January 16.

## Discussion and conclusions

In recent decades, the incidence of bacterial meningitis in Europe and North America has decreased due to widespread vaccination against *Haemophilus influenzae*, *Neisseria meningitidis*, and *S. pneumoniae* [3]. During the COVID-19 pandemic, hospital admissions for meningitis have decreased, probably due to the preventive hygiene measures (e.g., masks, hand washing) introduced in this period [4]. Prior to the pandemic, we admitted an average of two patients per year to our ICU for bacterial meningitis; by contrast, we did not admit any patients for this condition during the pandemic, which explains why two cases in a single week caught our attention.

Although several reports have described otitis media [5, 6] and meningitis [7–10] in patients with COVID-19, to our knowledge, no cases of bacterial meningitis secondary to otitis media in these patients have been described to date. We believe that the development of two such cases in such a short time span is highly unlikely to be coincidental, especially given that involvement of the omicron variant of SARS-CoV-2 is biologically plausible in both of these cases. Importantly, this variant was only confirmed by NGS in one of the two cases. We were unable to confirm the variant in the second patient due to the low viral load in the nasopharyngeal sample obtained at admission. However, the scant involvement of the lower respiratory tract, together with the time of diagnosis (i.e., during the peak spread of the omicron variant), strongly suggest the presence of the omicron variant.

The omicron variant was first identified in South Africa in November 2021. By January 2022, it had become the dominant variant in Europe. This variant seems to replicate less in the lungs than other variants [11], which would explain the lower incidence of acute respiratory failure in affected patients. However, this variant is also known to produce more symptoms in the upper respiratory tract, as evidenced by the two symptoms (sore throat, hoarse voice) that are consistently more prevalent in patients infected with this variant [2]. As we discuss below, involvement of the upper respiratory tract promotes the development of otitis media.

Acute otitis media is characterized by the presence of fluid in the middle ear and signs and symptoms of acute infection [12]. Viruses facilitate bacterial infection while the causative microorganisms are the colonizing bacteria typically found in the oropharynx (*S. pneumoniae* in 25% of healthy adults) [13]. Viral infections in the form of the “common cold” induce inflammation in the nasopharynx and the Eustachian tube, thus promoting bacterial adherence, colonization, and multiplication. Eustachian tube dysfunction causes negative pressure in the middle ear, which facilitates the migration of bacteria from the oropharynx into this region, leading to inflammation and infection. Suppurative otitis media can be complicated by acute mastoiditis, meningitis, and/or abscess [14].

This case report has several limitations. In one of the patients, we were unable to identify the causative variant, although the available evidence leads us to believe that it was the omicron variant. In addition, although we have not established a causal association between the virus and bacterial infection, we believe that the suggested pathophysiological mechanism explains this association well.

In conclusion, our data suggest that the omicron variant of the SARS-CoV-2 virus may promote the development of otitis media and secondary acute bacterial meningitis. We believe that it is important for clinicians to be aware of this possible association, which may facilitate early diagnosis and treatment. Undoubtedly, it will be interesting to monitor the presentation of any new variants of SARS-CoV-2 that emerge in the future.

## Data Availability

Data sharing is not applicable to this article as no datasets were generated or analyzed for this case report.

## Abbreviations

ICU: Intensive care unit
RT-PCR: Real time–polymerase chain reaction
GCS: Glasgow coma scale
CT: Computerized tomography
CSF: Cerebrospinal fluid
ME: Middle ear
EAC: External auditive conduct
M: Mastoid
